# Supplementary material for: Systematic characterization of existing and novel inducible transgenic systems in human pluripotent stem cells after prolonged differentiation
Source: bioRxiv. 2025 Nov 24:2025.10.17.683097. Preprint. [Version 2] doi: 10.1101/2025.10.17.683097 (PMC12653992; doi:10.1101/2025.10.17.683097)

bioRxiv preprint doi: https://doi.org/10.1101/2025.10.17.683097; this version posted November 24, 2025. The copyright holder for this preprint (which was not certified by peer review) is the author/funder, who has granted bioRxiv a license to display the preprint in perpetuity. It is made available under aCC-BY 4.0 International license.

SUPPLEMENTARY FIGURE LEGENDS

Figure S1: Effects of promoter replacement on pSBtet transposons in hPSCs and hPSC differentiation. (A,B) Western blot analyses of HEK293Ts transfected with pSBtet-GP-TF (panel A) and pSBtet-RP-TF (panel B). Panel A shows blot results for TAL1, with arrows indicating bands corresponding to the expected molecular weight of TAL1 (35-40kDa). Panel B shows blot results for KLF2, with arrow indicating dox-specific bands. As with Figure 1, dox doses are 0.75 and 7.5ug/mL. **(C)** Flow cytometry analysis of hPSCs transfected with plasmids expressing EGFP under control of RPBSA, CAG, PGK and CMV promoters. GFP+ cells are shown in blue, with the proportions indicated above the GFP+ gates. **(D)** Schematic of promoter pSBtet promoter replacement strategy. **(E,F)** Brightfield (top) and GFP (bottom) channels of hPSCs with stable integrations of pSBtet-GP-EF1A (panel E) and pSBtet-RP-EF1A (panel F). Ratios above images indicate transfection mass ratios of SB100X transposase and pSBtet transposons, with higher ratios improving integration efficiency. Scale bars are 500µm. **(G,H)** Brightfield and GFP channels from images of pSBtet-GP-EF1A hPSCs at days 5 (panel G) and 10 (panel H) of hMGL differentiation. Puro-induced cell death is visible at day 10 but not day 5. Scale bars are 500µm.

Figure S2: Quantification of leaky expression in Tet-On and tTS-based systems. (A) Geometric mean fluorescence intensity (MFI) at 488nm of all singlet gated hPSCs (left) and the percentage of EGFP+ cells when gated at ~0.9% against unengineered WT cells, all in the absence of doxycycline (DOX). **(B)** Time course of EGFP expression response represented in hours post-DOX exposure in hPSCs engineered with the standard third-generation Tet-On system, TetO3G.

Figure S3: Quantification of leaky expression in DHFR degron systems. (A) Dose-response of EGFP expression represented 24h post-trimethoprim (TMP) exposure in hPSCs engineered with all DHFR degron systems. **(B)** Geometric mean fluorescence intensity (MFI) at 488nm of all singlet gated hPSCs (left) and the percentage of EGFP+ cells when gated at ~0.9% against unengineered WT cells, all in the absence of TMP.

Figure S4: X^{on}-EGFP performance in alternative hPSCs and hMGLs generated with a different differentiation protocol. (A-B) hPSCs from a different genetic background as those in **Fig. 4** display tunable LMI070-mediated EGFP expression. **(C)** hMGLs produced from hPSCs shown in panel A, but with a different differentiation protocol[66] than those shown in Fig. 4, display LMI070 dose-dependent EGFP expression, with no obvious toxicity.

Figure S5: Quantification of leaky expression in X^{on} system. (A) Representative flow cytometry plot demonstrating distribution of 488nm fluorescence in unengineered WT hPSCs (left), overlayed with X^{on}-EGFP-engineered hPSCs (right), both with ~0.9% stringency gate illustrated. **(B)** Geometric mean fluorescence intensity (MFI) at 488nm of all singlet gated X^{on}-EGFP hPSCs (left) and the percentage of EGFP+ cells when gated at ~0.9% against unengineered WT cells, all in the absence of LMI070.

Figure S6: Approaches to ameliorate technical limitations of the X^{on} system. (A) Western blot analysis of AAVS1-targeted X^{on}-SALL1 in hPSCs from a different genetic background than those shown in Fig. 5A, indicating leakiness and poor inducibility in the presence of LMI070. **(B)** Western blot analysis of HEK293Ts transfected with codon-optimized X^{on}-SALL1 and Met→Ile mutated X^{on}-EGFP-P2A-TREM donor constructs. **(C)** Western blot analysis of HEK293Ts transfected with X^{on}-EGFP-P2A-SALL1 donor construct. **(D)** Western blot analysis of HEK293Ts transfected with X^{on}-TREM2 WT, X^{on}-TREM2 R47H, and X^{on}-EGFP-P2A-TREM2 donor

bioRxiv preprint doi: https://doi.org/10.1101/2025.10.17.683097; this version posted November 24, 2025. The copyright holder for this preprint (which was not certified by peer review) is the author/funder, who has granted bioRxiv a license to display the preprint in perpetuity. It is made available under aCC-BY 4.0 International license.

constructs. **(E)** Western blot analysis of HEK293Ts transfected with X^{on}-P2A-SALL1 and Met→Ile mutated X^{on}-EGFP-P2A-SALL1 donor constructs.

bioRxiv preprint doi: https://doi.org/10.1101/2025.10.17.683097; this version posted November 24, 2025. The copyright holder for this preprint (which was not certified by peer review) is the author/funder, who has granted bioRxiv a license to display the preprint in perpetuity. It is made available under aCC-BY 4.0 International license.

Figure S1

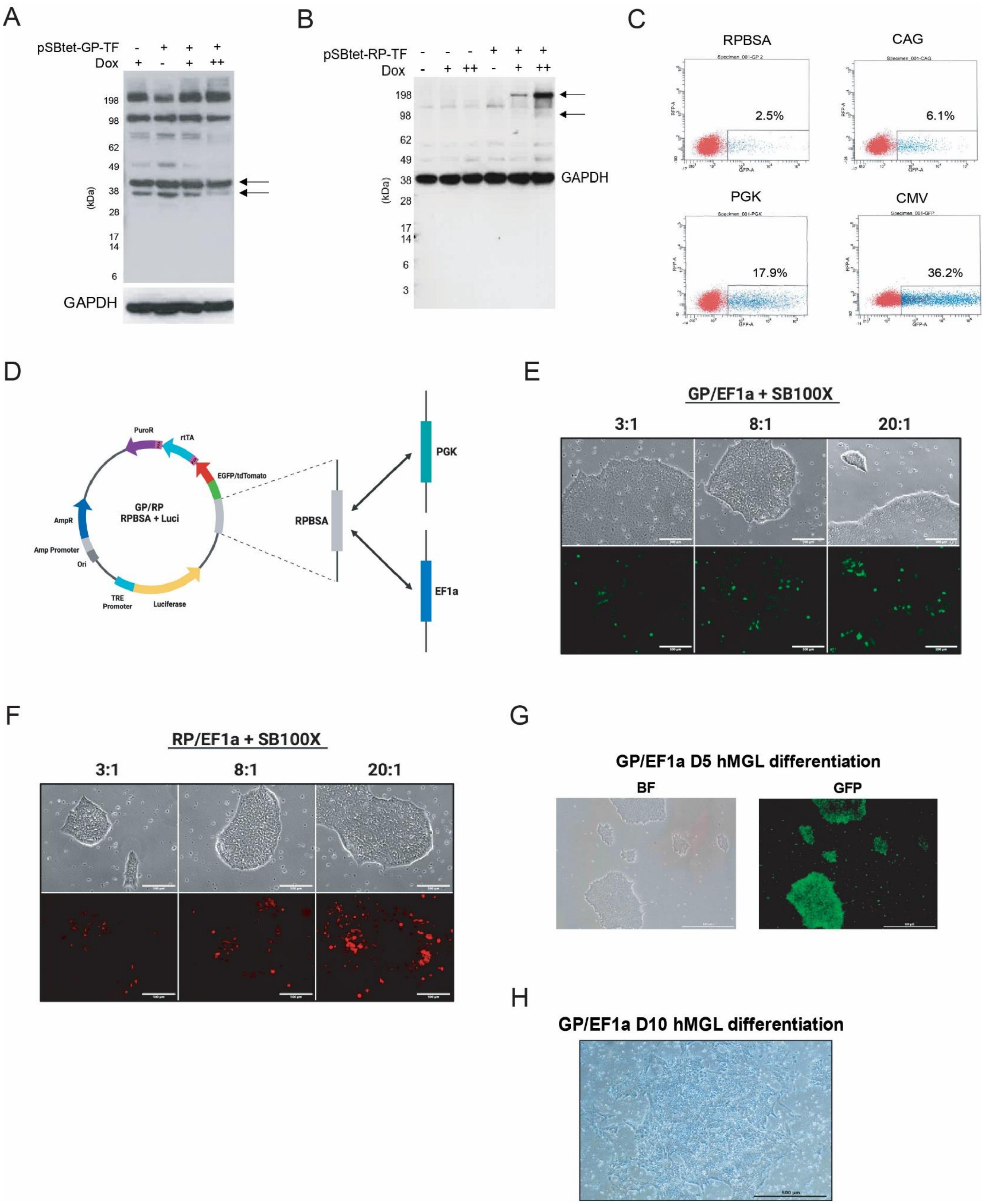

bioRxiv preprint doi: https://doi.org/10.1101/2025.10.17.683097; this version posted November 24, 2025. The copyright holder for this preprint (which was not certified by peer review) is the author/funder, who has granted bioRxiv a license to display the preprint in perpetuity. It is made available under aCC-BY 4.0 International license.

Figure S2

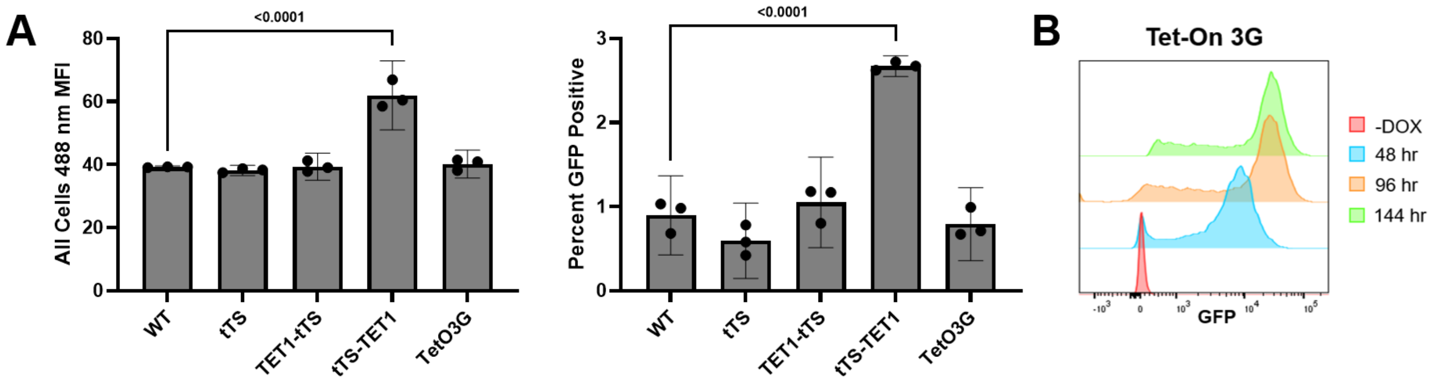

bioRxiv preprint doi: https://doi.org/10.1101/2025.10.17.683097; this version posted November 24, 2025. The copyright holder for this preprint (which was not certified by peer review) is the author/funder, who has granted bioRxiv a license to display the preprint in perpetuity. It is made available under aCC-BY 4.0 International license.

Figure S3

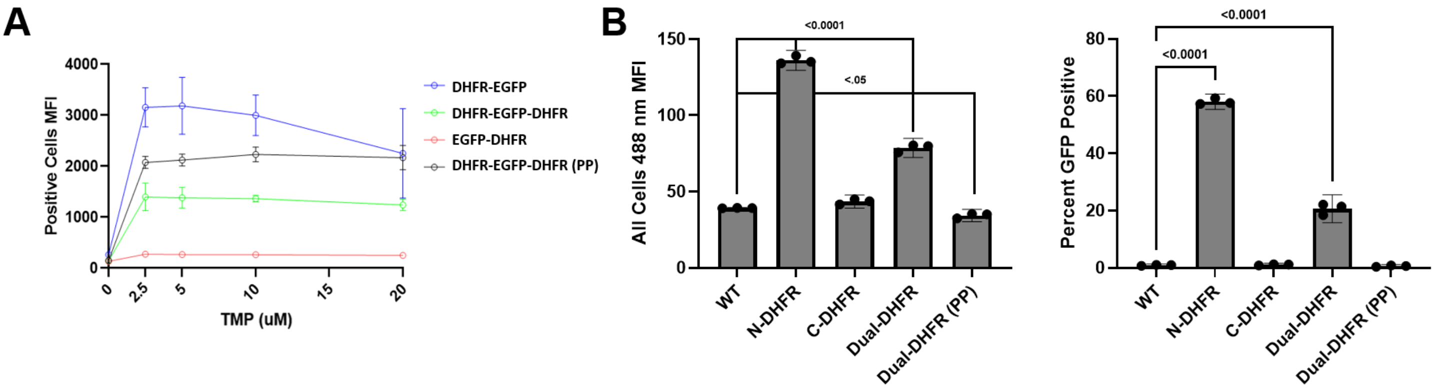

bioRxiv preprint doi: https://doi.org/10.1101/2025.10.17.683097; this version posted November 24, 2025. The copyright holder for this preprint (which was not certified by peer review) is the author/funder, who has granted bioRxiv a license to display the preprint in perpetuity. It is made available under aCC-BY 4.0 International license.

Figure S4

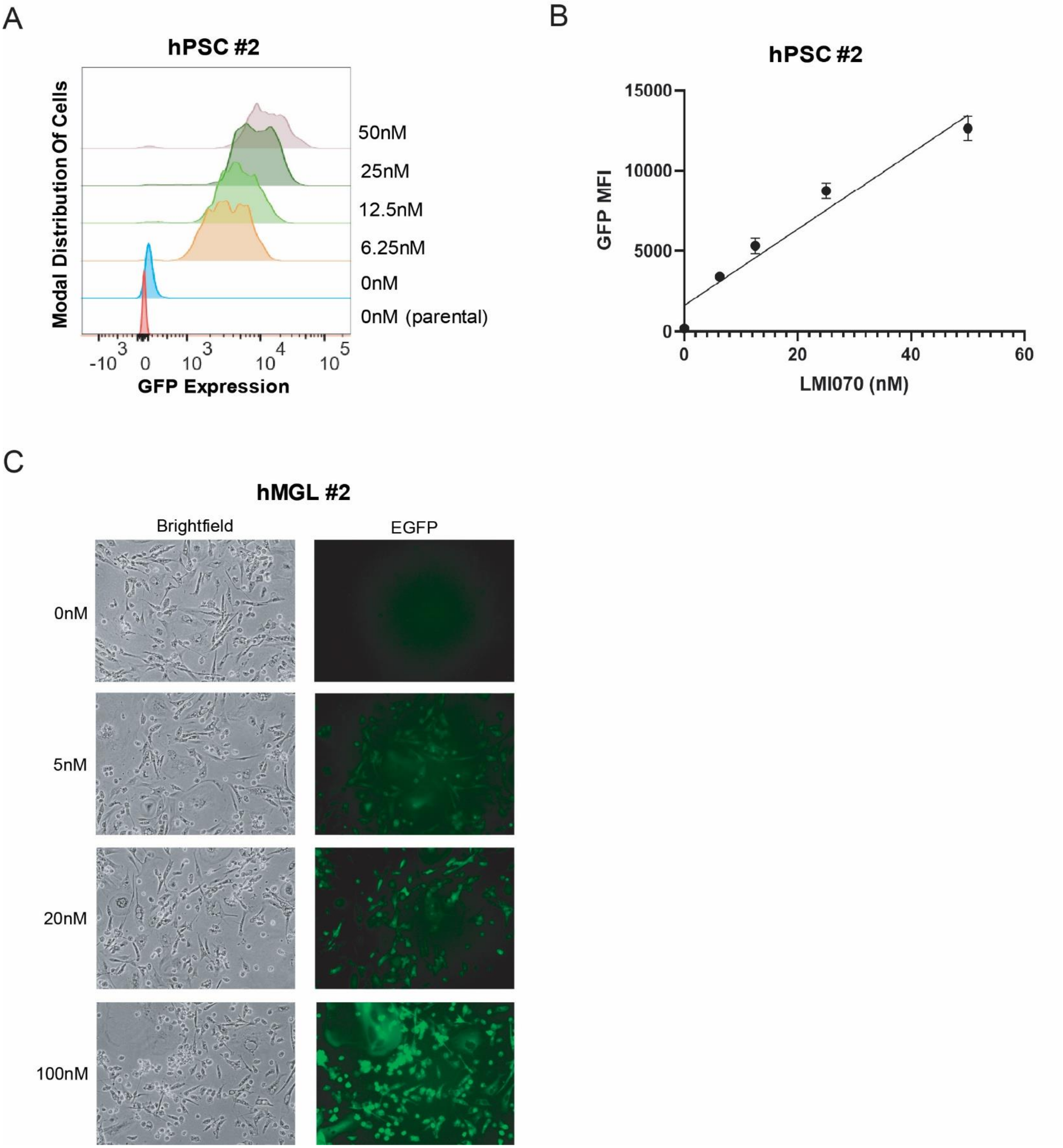

bioRxiv preprint doi: https://doi.org/10.1101/2025.10.17.683097; this version posted November 24, 2025. The copyright holder for this preprint (which was not certified by peer review) is the author/funder, who has granted bioRxiv a license to display the preprint in perpetuity. It is made available under aCC-BY 4.0 International license.

Figure S5

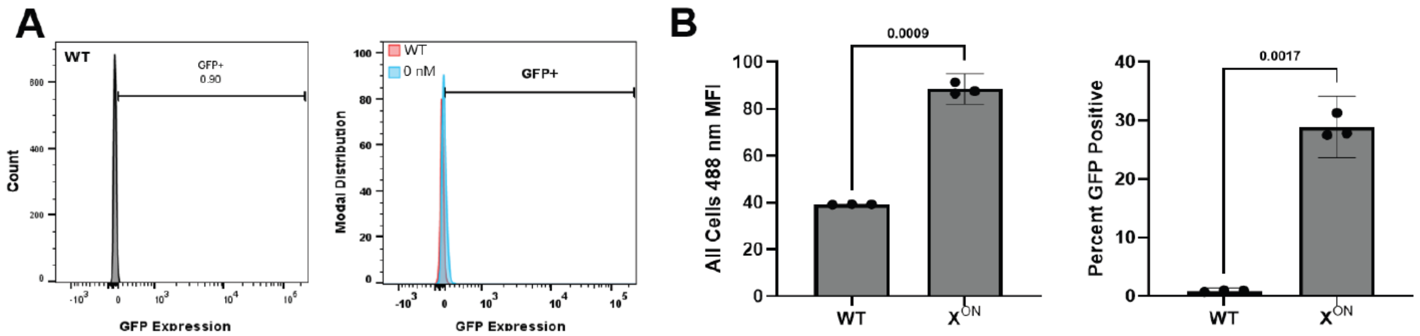

bioRxiv preprint doi: https://doi.org/10.1101/2025.10.17.683097; this version posted November 24, 2025. The copyright holder for this preprint (which was not certified by peer review) is the author/funder, who has granted bioRxiv a license to display the preprint in perpetuity. It is made available under aCC-BY 4.0 International license.

Figure S6

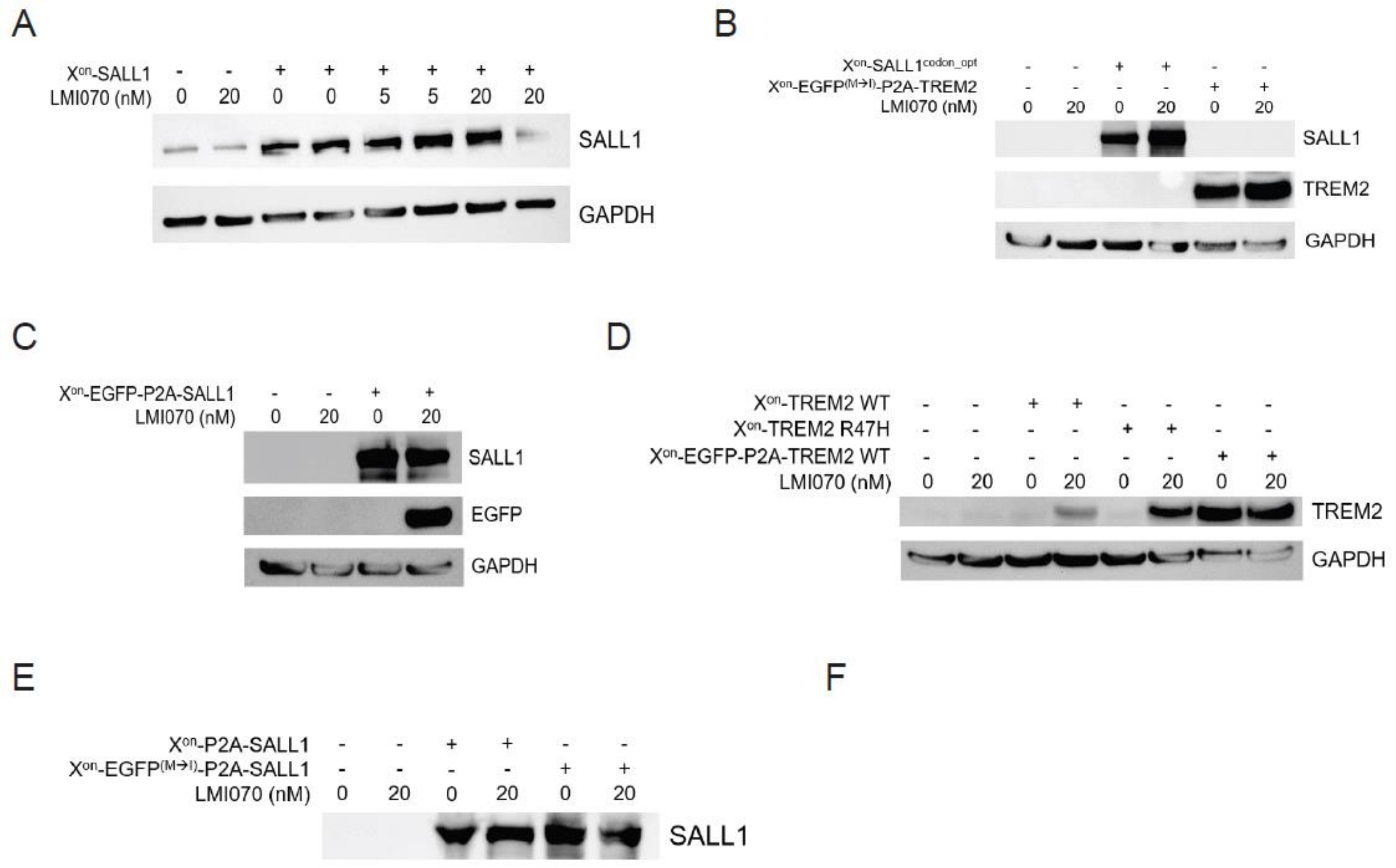

Supplement: 1 [file NIHPP2025.10.17.683097v2-supplement-1.pdf]
